# Advances in CT-based lung function imaging for thoracic radiotherapy

**DOI:** 10.3389/fonc.2024.1414337

**Published:** 2024-09-02

**Authors:** Suyan Bi, Qingqing Yuan, Zhitao Dai, Xingru Sun, Wan Fatihah Binti Wan Sohaimi, Ahmad Lutfi Bin Yusoff

**Affiliations:** ^1^ School of Medical Sciences, Universiti Sains Malaysia, Kelantan, Malaysia; ^2^ National Cancer Center/National Clinical Research Center for Cancer/ Cancer Hospital & Shenzhen Hospital, Chinese Academy of Medical Sciences and Peking Union Medical College, Shenzhen, China; ^3^ Huizhou Third People’s Hospital, Guangzhou Medical University, Huizhou, Guangdong, China; ^4^ Department of Nuclear Medicine Radiotherapy and Oncology, School of Medical Sciences, Universiti Sains Malaysia, Kelantan, Malaysia

**Keywords:** CT-based functional imaging (CTVI), ventilation imaging, perfusion imaging, radiotherapy, four-dimensional CT(4D-CT), magnetic resonance imaging (MRI), single-photon emission computed tomography (SPECT)

## Abstract

The objective of this review is to examine the potential benefits and challenges of CT-based lung function imaging in radiotherapy over recent decades. This includes reviewing background information, defining related concepts, classifying and reviewing existing studies, and proposing directions for further investigation. The lung function imaging techniques reviewed herein encompass CT-based methods, specifically utilizing phase-resolved four-dimensional CT (4D-CT) or end-inspiratory and end-expiratory CT scans, to delineate distinct functional regions within the lungs. These methods extract crucial functional parameters, including lung volume and ventilation distribution, pivotal for assessing and characterizing the functional capacity of the lungs. CT-based lung ventilation imaging offers numerous advantages, notably in the realm of thoracic radiotherapy. By utilizing routine CT scans, additional radiation exposure and financial burdens on patients can be avoided. This imaging technique also enables the identification of different functional areas of the lung, which is crucial for minimizing radiation exposure to healthy lung tissue and predicting and detecting lung injury during treatment. In conclusion, CT-based lung function imaging holds significant promise for improving the effectiveness and safety of thoracic radiotherapy. Nevertheless, challenges persist, necessitating further research to address limitations and optimize clinical utilization. Overall, this review highlights the importance of CT-based lung function imaging as a valuable tool in radiotherapy planning and lung injury monitoring.

## 1 Introduction

Lung cancer is one of the most common malignancies worldwide and ranks among the highest in morbidity and mortality in most countries. Radiation therapy, a crucial treatment modality, effectively inhibits tumor growth and dissemination (1). However, traditional radiotherapy administers identical dosing priority and avoidance protocols to all normal lung tissues, neglecting the functional disparities among various regions. Radiation-induced damage to normal lung tissue frequently results in complications, such as acute radiation pneumonia, substantially deteriorating patients’ quality of life (2). Minimizing damage to normal lung tissue while enhancing therapeutic outcomes during radiotherapy has emerged as a central focus in clinical research. Studies (3–8) have highlighted Dmean, %V5Gy, %V20Gy, and %V30Gy (%VxGy, represents the percentage of lung volume receiving a radiation dose of at least x Gy) as pivotal metrics for assessing Radiation-Induced Pulmonary Injury (RIPI). Lung function imaging, a technology utilizing diverse imaging modalities to evaluate the structural and functional integrity of the lungs, facilitates quantitative assessment of lung function and precise delineation of functional regions (9–11). The advantages of utilizing lung function imaging in radiotherapy are twofold: firstly, it facilitates personalized treatment planning by directing radiation reaching the tumor through lung areas with poor gas exchange function, thus reducing the risk of damage to healthy lung tissue (12); secondly, it facilitates more precise and prompt evaluation of pulmonary injury, as well as enabling real-time monitoring of lung function. This capability is extensively utilized in diagnosing and prognostic assessment of both acute and chronic lung conditions (13–15).

In the past decade, significant advancements in medical imaging technologies have profoundly transformed the evaluation of lung function, notably with the advent of CT-based lung ventilation function imaging (CTVI). CTVI involves delineating lung function across various regions, yielding detailed ventilation maps that capture differences in lung ventilation between phase-resolved four-dimensional CT (4D-CT) scans and end-inspiratory and end-expiratory CT images (16). This innovative approach enables the extraction of functional parameters like lung volume and ventilation distribution, providing a comprehensive assessment and characterization of lung function. Furthermore, CTVI facilitates the quantitative description of both physiological and pathological states of lung tissue (17), providing valuable insights into lung health.

Although several CTVI reviews have been published, most of them date back five years (18–20), excluding recent research advancements. Moreover, certain reviews focused solely on perfusion function imaging or machine learning (21, 22), failing to provide a comprehensive overview of CTVI’s technical advancements. In fact, CTVI research holds significant importance for radiotherapy. For instance, CT-based lung function studies eliminate the need for extra scans during radiotherapy, thereby reducing patient exposure to radiation and costs. This represents a distinct advantage over other imaging modalities. As a result, the aim of the present paper was to bridge these gaps by addressing the progress of CTVI research methods and the clinical application in radiotherapy in recent decades. Additionally, this review will discuss the limitations of CTVI compared to other lung function imaging modalities, as well as any changes in the theoretical basis and evaluation criteria of research. Our analysis aims to stimulate further research endeavors and offer valuable insights to enhance the utilization of CT-based lung function imaging in radiotherapy.

## 2 Methods

In this review, we meticulously explored the potential and challenges associated with CT-based lung function imaging in the context of radiotherapy. Leveraging WOS and PubMed databases, we conducted an extensive literature search spanning from 2000 to 2024, utilizing Boolean logic (CTVI or “CT ventilation imaging” or “function imaging”or “functional lung”)AND (radiotherapy or “radio oncology”or “Thoracic radiotherapy”) resulting in the retrieval of 202 articles meeting our criteria. This meticulous approach facilitated the identification of pertinent literature aligning with the scope of this review.

Two reviewers (S.B. and Q.Y.) extracted the appropriate information from each study independently. Article citations included, but were not limited to, techniques, publication year, imaging modalities, ventilation or perfusion, study population size, research types and aims, clinical effect. Contradictions were discussed with two reviewers (Z.D and X.S.). According to our research objective, total of 77 articles were finally included with 10 reviews and two books. 61 articles of them were published in recent decade.

## 3 Results

Studies investigating CT lung function imaging can be broadly categorized into three key areas. Firstly, the development of CTVI extraction technology, as documented in (23–25). This advancement focuses on deriving variable values for ventilation-related parameters, utilizing selection algorithms grounded in deformable image registration (DIR) techniques and alternative methodologies. Secondly, the quantitative analysis of CTVI, as reported in (25–27), which is based on assessing the magnitude of changes in pertinent images, involves seeking suitable tools, including machine learning, modeling, and statistical approaches. Lastly, with respect to clinical applications, radiotherapy strategies utilizing CTVI, as detailed in (25, 28–30), encompass dose allocation optimization, prediction of adverse effects, and monitoring of treatment outcomes.

### 3.1 The extraction methods of CTVI

#### 3.1.1 Deformable image registration based selection algorithms

Existing computed tomography (CT) ventilation imaging methods primarily evolved from image processing research, DIR of 4DCT lung scans. Subsequent quantification involves assessing local breathing-induced variations in volume, Jacobian, and Hounsfield Units (HU) (10, 23, 25). Zhang et al. (31) initially introduced an algorithm that utilizes a direct geometrical approach to estimate ventilation based on lung volume changes (ΔV). This approach derives specific volume changes by computing the volume of deformed elements, considering eight vertex positions of a voxel, which are transformed using DIR. The volume of each tetrahedron is calculated using the following formula:


(1) $$V=\left(\vec{b}-\vec{a}\right)\cdot \left[\left(\vec{c}-\vec{a}\right)\times \left(\vec{d}-\vec{a}\right)\right]/6$$

where $\vec{a}$ , $\vec{b}$ , $\vec{c}$ and $\vec{d}$ are the vertices of the tetrahedron as vectors. Summing the volumes of the six tetrahedrons from the DIR deformation matrix yields the volume of a given polyhedron.

In addition to the direct geometrical method, Reinhardt et al. (32) proposed an approximate change in volume of voxels method by calculating the Jacobian of the deformation field. The Jacobian matrix, based on the theory that the local partial derivatives of the deformation field are all related to the volume change of voxels in a given lung tissue, describes the local volume change caused by ventilation (25). The calculation of the Jacobian relies exclusively on the DIR transformation function and the Jacobian determinant derived from the deformation field between different breathing phases, as obtained through image registration. The formula is expressed as follows (33):


(2) $${\text{Vent}}_{exh}^{Jac}(p)\left|\begin{array}{ccc} 1+\frac{\partial d_{p}^{1}}{\partial x_{1}} & \frac{\partial d_{p}^{1}}{\partial x_{2}} & \frac{\partial d_{p}^{1}}{\partial x_{3}}\\ \frac{\partial d_{p}^{2}}{\partial x_{1}} & 1+\frac{\partial d_{p}^{2}}{\partial x_{2}} & \frac{\partial d_{p}^{2}}{\partial x_{3}}\\ \frac{\partial d_{p}^{3}}{\partial x_{1}} & \frac{\partial d_{p}^{3}}{\partial x_{2}} & 1+\frac{\partial d_{p}^{3}}{\partial x_{3}} \end{array}\right|$$

Where $d_{p}^{1}$ , $d_{p}^{2}$ and $d_{p}^{3}$ represent the displacement components of pixel points along the left and right, abdomen and back, and head and foot directions respectively. ${\text{Vent}}_{exh}^{Jac}(p)$ values between 0 and 1 indicate reduced lung volume, ${\text{Vent}}_{exh}^{Jac}(p)=1$ indicates no change in the volume of this area, and ${\text{Vent}}_{exh}^{Jac}(p)>1$ indicates volume expansion of pixels.

Apart from the Jacobian algorithm, the CT value method offers an alternative approach to estimating the distribution of pulmonary ventilation (28). This method utilizes variations in CT values during the transition from inhalation to exhalation to approximate lung ventilation, as the magnitude of CT values is intimately correlated with lung tissue density. The formula for ventilation capacity relating to the maximum expiratory and inspiratory phases is expressed as follows (24):


(3) $${\text{Vent}}_{\text{exh}}^{\text{Hu}}(p)=\frac{{\text{HU}}_{\text{exh}}\left({\text{X}}_{\text{P}}\right)\times {\text{G}}_{\text{k}1}-{\text{HU}}_{\text{inh}}\left({\text{x}}_{\text{p}+{\text{d}}_{\text{p}}}\right)\times {\text{G}}_{\text{K}1}}{{\text{HU}}_{\text{inh}}\left({\text{x}}_{\text{p}+{\text{d}}_{\text{p}}}\right)+1000\;}\times {\text{G}}_{\text{K}2}$$

Where ${\text{HU}}_{\text{exh}}\left({\text{x}}_{\text{p}}\right)$ represents the CT value of each pixel in the image corresponding to the maximum expiratory phase; ${\text{HU}}_{\text{inh}}\left({\text{x}}_{\text{p}+{\text{d}}_{\text{p}}}\right)$ represents the CT value of pixel p in the image corresponding to the maximum inspiratory phase after the action of the displacement vector; ${\text{G}}_{\text{k}1}$ and G_k2_ represent Gaussian filters for image smoothing and denoising.

To evaluate the advantages and disadvantages of these three approaches, Castillo et al. (23) conducted a comparative study and found that although the Jacobian-based approach was more widely used, the correlation between ventilation function based on changes in CT values and clinical references was higher. Latififi (34) and J Cai, et al. (35) compared the above three ventilation imaging algorithms and found that the similarity between ΔV and Jacobian is higher than that between HU and Jacobian, and ΔV and HU. Nonetheless, the accuracy of the DIR-based method is heavily influenced by the precision of image registration. Furthermore, despite the high consistency of the results obtained from the ΔV and Jacobian methods, the absence of a standard functional area for comparison in this study renders it impossible to definitively state that the lung function area derived from these methods is superior in terms of accuracy.

#### 3.1.2 No-DIR selection methods

In addition to relying on DIR for obtaining image shapes at different positions, some improved methods, not reliant on image registration also yield promising results. Szmul (36) and Xue Peng (37) combined superpixel segmentation with 4DCT image registration methods to calculate pulmonary ventilation distribution. Hegi-Johnson et al. (38) estimated blood-gas exchange based on a time-averaged 4DCT Hounsfield unit (HU) value and pulmonary ventilation based on the product of air and tissue density fraction in all phases of a respiratory cycle; Li M et al. (39) proposed an improved DIR method that combined the variable intensity flow (VIF) block matching algorithm with the finite element method (FEM) to evaluate lung deformation from the end of expiration to the end of inspiration, thereby improving registration accuracy. In contrast to traditional CT value-based and Jacobian methods, This new approach exhibits smoother characteristics and provides a more accurate representation of regional variations in lung ventilation.

With significant advancements in scanning speed and imaging resolution, a noninvasive lung ventilation assessment method has been devised, leveraging the wash-in and/or wash-out rates of the nonradioactive gas xenon. This approach produces color-coded images of regional ventilation, enabling comprehensive analysis of ventilation patterns and fusion with CT imaging. In recent studies leveraging the latest CT technology, Honda et al. (40) evaluated the single-breath-hold technique for ventilation mapping, employing a dual-energy CT scanner. Another study concentrated on biomechanics-based image registration and advanced air segmentation methods to generate 4DCT ventilation maps (41). These new techniques have demonstrated high accuracy and the potential to provide a more precise characterization of ventilation distribution.

The improved-DIR method has been found to effectively mitigate uncertainties stemming from image registration. Furthermore, the incorporation of novel technologies in this domain, which have demonstrated encouraging outcomes, for instance, Xenon-enhanced images displayed superior image quality upon visual assessment, holds significant potential for future applications. Nonetheless, the clinical implementation of radiotherapy encounters obstacles, primarily due to the requirement for novel devices and the current lack of prospective trials.

### 3.2 Quantitative analysis in CTVI

In the study of lung ventilation function, it is crucial to acquire change values of ventilation function at varying sampling volumes and subsequently conduct image segmentation and visualization utilizing these alterations. Typically, the conventional imaging process involves image deformation registration or density and gray value change to obtain the change matrix, followed by image segmentation (23, 24, 38). This part pertains to the quantitative analysis methodologies of image segmentation.

Image segmentation tasks can be classified into two categories according to different processing purposes: semantic segmentation and instance segmentation (42). Semantic segmentation involves pixel-level classification, assigning corresponding categories to all pixels in an image. In contrast, instance segmentation differentiates individual objects within the same category, utilizing the information obtained from semantic segmentation. Designing segmentation methods to distinguish organ or lesion pixels requires task-specific image data to provide critical details. For medical imaging modalities, the data sources can be X-ray, CT, and MRI. Edge detection, template matching techniques, region growing, graph cuts, active contour lines, machine learning, and other mathematical methods were the main approaches to medical image segmentation in the early days (43–45).

#### 3.2.1 Deep learning was used in quantitative analysis

In recent years, deep learning (DL) has advanced significantly and found applications in medical imaging processes (24). In CTVI, the primary approach for machine learning and deep learning entails utilizing labeled ventilation and perfusion function image data as training targets, with radiological features extracted from conventional CT images serving as training inputs. Utilizing machine learning and deep learning, functional predictive models are developed to automate the assessment and segmentation of distinct functional regions within CT images.

As a traditional DL approach, Convolutional Neural Networks (CNNs) are renowned for their superior performance and accuracy. They effectively implement feature representation extraction for images, obviating the necessity for manual feature engineering in image segmentation, thereby becoming the primary choice in this domain. Zhong et al. (46) developed a method utilizing deep CNNs to directly derive ventilation images from 4DCT, bypassing the need for explicit image registration. The initial convolutional layer comprised 32 kernels, followed by eight additional convolutional layers, all incorporating the ReLU activation function. They found that deep CNNs excel in generating ventilation imaging, effectively mitigating uncertainties as compared to conventional 4DCT deformable registration methods.

In addition to CNNs, Long et al. (47) proposed fully convolutional networks (FCN) learning method. This method applies several convolutional blocks consisting of convolution, activation, and pooling layers on the encoder path to capture semantic representation. Inspired by the architecture of FCNs and encoder-decoder models, some researches (48–50) developed the U-Net model for biomedical image segmentation, tailored for practical use in medical image analysis that is applicable across various imaging modalities. Later, Liu (51) devised a DL method based on U-Net for producing 4DCT ventilation imaging. The accuracy of DL-based ventilation imaging was evaluated against SPECT ventilation imaging (SPECT-VI) by comparing the density change-based and Jacobian-based methods. The findings indicated that the DL-based method surpassed other approaches in terms of performance.

As artificial intelligence progresses, lung function imaging leveraging deep learning holds significant potential for advancements. Within the realm of deep learning, the development of a unified standard for lung function image selection represents an important research direction.

### 3.3 Radiotherapy strategies based on CTVI

Currently, the application of lung function imaging based on CT in radiotherapy has yielded promising results. Studies have indicated that CTVI can offer more accurate anatomical and functional information for designing radiation therapy plans, effectively minimizing dose exposure to normal tissues (25). Additionally, lung function imaging can aid in predicting side effects and monitoring therapeutic effects, enabling personalized radiation therapy. As shown in **Table 1**, references (9, 29, 30, 32, 52–56) have demonstrated that radiotherapy schedules avoiding functional lung can reduce adverse reactions to thoracic radiotherapy, while references (12, 37, 57–60) have focused on whether functional lung dose index can better evaluate and predict radiation lung injury.

**Table 1:** Study on clinical application of CTVI.

Author	Years	Case	Treatment techniques	Research type	Application
Takemoto S, et al. (12)	2021	70	Photon therapy, SBRT	prospective	Evaluation and prediction
Yamamoto T, et al (24),	2018	14	Photon therapy, IMRT	prospective	Functional lung avoidance
Li S, et al. (25)	2023	17	Photon therapy, TomoTherapy (HT)	retrospective	Functional lung avoidance
Huang YH,et al (27)	2022	15	Photon therapy	retrospective	Functional lung avoidance
Waxweiler, et al. (48)	2017	96	Photon therapy, 3D-CRT or IMRT	retrospective	Functional lung avoidance
Vinogradskiy, et al. (49)	2022	67	Photon therapy,IMRT	prospective	Functional lung avoidance
Huang Q, et al. (50)	2018	8	proton therapy, DSPT, IMPT	retrospective	Functional lung avoidance
Ieko Y,et al (51)	2020	13	proton therapy, SBPT	retrospective	Functional lung avoidance
Dougherty JM, et al. (25)	2021	31	proton therapy, IMPT	retrospective	Functional lung avoidance
Vinogradskiy Y, et al (52)	2013	96	Photon therapy, 3D-CRT or IMRT	retrospective	Evaluation and prediction
Vinogradskiy Y, et al (53)	2022	6	Photon therapy	retrospective	Evaluation and prediction
Farr KP, et al (54)	2015	58	Photon therapy,IMRT	retrospective	Evaluation and prediction
Lan F, et al. (55)	2016	37	Photon therapy	retrospective	Evaluation and prediction
Patton TJ, et al. (56)	2018	12	Photon therapy	retrospective	Evaluation and prediction
Faught AM, et al. (57)	2017	70	Photon therapy	retrospective	Evaluation and prediction

#### 3.3.1 Functional lung avoidance radiotherapy/functional planning design

**Figure 1:**
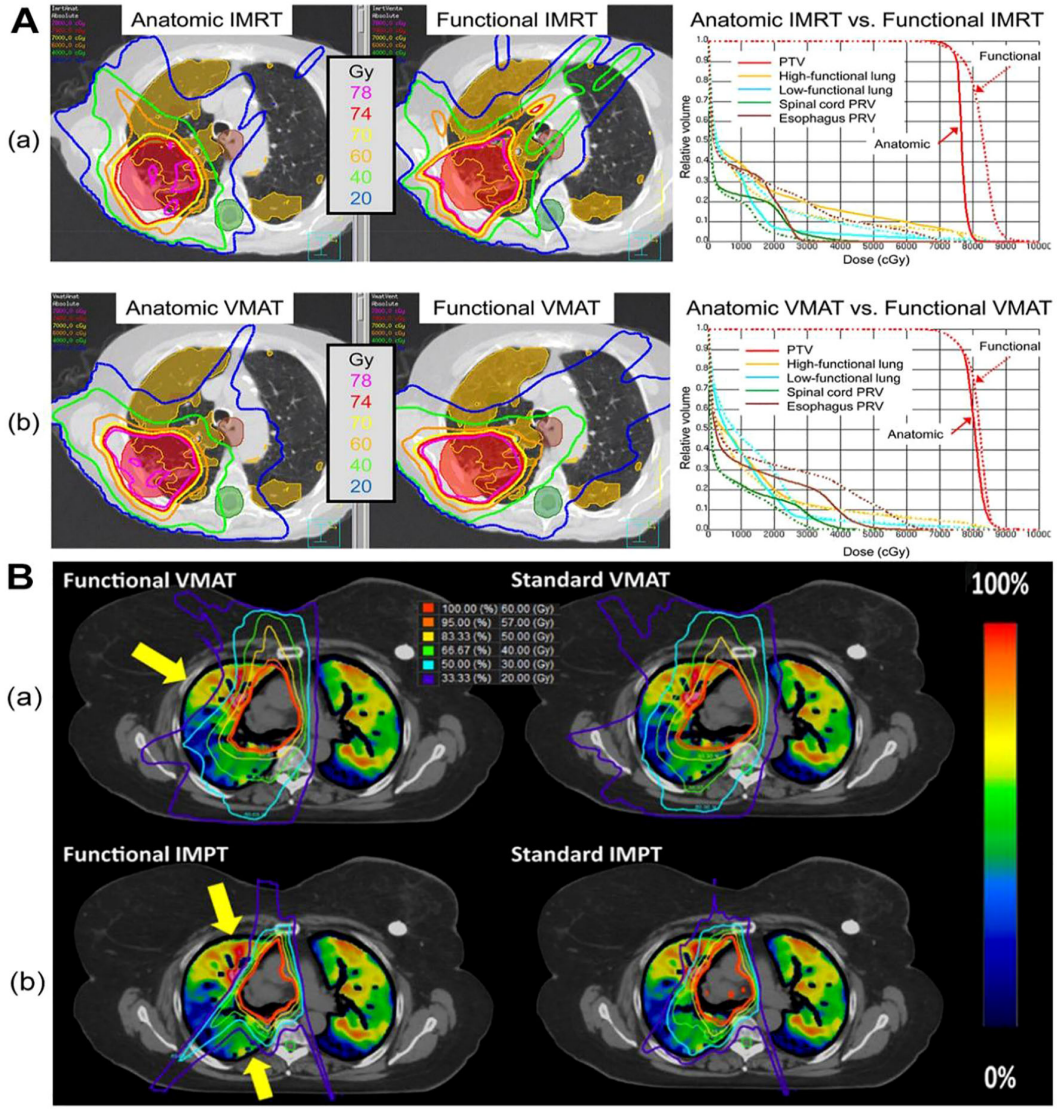
The dose distribution in CTVI-based planning of photon and proton therapy respectively. Figure1 is the comparison of a functional avoidance plan and non-functional plan. The CT, CT-ventilation images, isodose lines, and PTV (shown in red) are presented for both groups of plans of photon **(A)** and proton **(B)** herapy. The arrows highlight the regions with the most prevalent functional lung sparing. A printed with permission from Yamamoto T, et al (25) and B printed with permission from Dougherty JM, et al (56).

In 2007, Yaremko et al. (28) embarked on integrating lung ventilation data into the NSCLC program. In recent decades, numerous subsequent clinical studies have affirmed that radiotherapy using 4DCT functional lung imaging aiming at avoiding functional lung can decrease the occurrence of adverse radiation reactions such as radiation pneumonia (25) (**Figure 1**). For example, Takemoto S et al. (12) investigated whether the decline in pulmonary function post-SBRT could be anticipated based on radiation dose-volume parameters. Their studies revealed correlations between planning target volume (PTV) and alterations in mean forced vital capacity (FVC), as well as associations between changes in predicted percent FVC and %V5Gy and %V40Gy. Yamamoto T (29) and Li S (30) researches also confirmed that lung functional image-guided radiation therapy, which avoids irradiating highly functional regions, has the potential to reduce pulmonary toxicity following RT. Despite ample evidence favoring the integration of lung function imaging in radiotherapy to mitigate pulmonary toxicity, the study conducted by Vinogradskiy et al. (53) merits attention. They performed a multi-institutional phase 2 clinical trial that utilized 4DCT ventilation function imaging in the planning of thoracic radiotherapy. In this study, comprising 67 patients, only 10 (14.9%) exhibited RP, with an upper limit of 95% CI at 24.0%, representing a statistically significant reduction compared to conventional radiotherapy. This study confirms that functional lung avoidance radiotherapy plans effectively mitigate the incidence of radiation pneumonitis (RP).

Functional lung imaging has not only been applied in photon therapy but also in proton therapy (PT). Huang (54) and Leko (55) designed functional proton plans using double scattering proton therapy (DSPT) and intensity-modulated proton therapy (IMPT) based on 4DCT ventilation images, comparing them with functional photon plans employing 3D-CRT, IMRT, and VMAT. Their studies revealed that DSPT and IMPT plans offered superior protection of the low-dose regions of the total lung (V5) compared to IMRT. Additionally, functional DSPT and functional IMPT exhibited marked advantages in preserving high-functioning lung tissue, outperforming anatomical planning approaches. Dougherty JM, et al. (56) also assessed the potential dosimetric gains of conducting functional avoidance-based proton treatment planning using 4DCT-derived ventilation imaging. They observed a mean 5.7% reduction in Grade 2+ RP with functional IMPT, with a 26% higher reduction in individual patients compared to standard IMPT planning (**Figure 1**).

These studies show that functional lung imaging, when applied to protect normal lung tissue, offers advantages not only in X-ray therapy but also in proton therapy. Furthermore, NTCP calculations indicated a further decrease in the risk of pulmonary complications when using functional IMPT.

#### 3.3.2 The prediction of side effects and the monitoring of therapeutic effects

Functional imaging integrated into radiotherapy has demonstrated greater advantages compared to traditional methods, enabling more accurate prediction through new functional LDVP, such as mean functional lung dose (f-MLD), functional lung V20 (fV20), etc. Vinogradskiy Y, et al. (53, 57, 58) sought to correlate 4DCT with chest adverse reactions post-radiotherapy. Their findings indicated that IMRT planning guided by ventilation function imaging was capable of decreasing the incidence of grade 2+ and 3+ radiation pneumonitis by 7.1% and 4.7%, respectively, as predicted by Normal Tissue Complication Probability models. They also concluded that dose-function index incorporating functional lung information holds greater significance in predicting radiation pneumonia than dose-volume index. Furthermore, Faught AM et al. (59) compared three different methods of dose-function metrics incorporating the patient’s 4D-CT ventilation image and treatment planning dose distribution (structure-based approaches, image-based approaches using the dose-function histogram, and nonlinear weighting schemes) to predict grade 3 or higher RP using the NTCP model. When the functional lung dose exceeded 20Gy, the prediction accuracy of the structure-based approaches was higher than the other two methods. Additional studies (60–62) further validated the predictive capability of 4D-CT for radiation pneumonitis (RP). Research findings demonstrate that implementing functional lung avoidance techniques can reduce the risk of developing severe radiation pneumonia (grade 3 or higher) by 18%, with some patients experiencing up to a 20% reduction. This suggests that functional lung avoidance combined with functional information can lower the incidence of thoracic adverse reactions after radiotherapy.

For prospective clinical trials that utilize CT-ventilation, such as NCT02528942 and NCT02843568, the outcomes of these trials serve as pivotal evidence for applying lung ventilation function in assessing and predicting RP in clinical settings. As an example, the phase II study (NCT025242) demonstrated that functional lung avoidance planning, grounded in 4D-CT ventilation imaging, could significantly decrease the occurrence of grade 2 or higher radiation pneumonitis by 14.9% among lung cancer patients. The comprehensive list of currently registered clinical trials investigating lung functional imaging-guided radiotherapy at ClinicalTrials.gov is summarized in **Table 2**.

**Table 2:** Registered clinical trials of CT-based functional-guided radio- therapy (from ClinicalTrials.gov).

NTC number	Status	Study Results	Conditions	Interventions	Locations
NCT05103670	Recruiting	No Results Available	• Pulmonary Embolism • Dyspnea	• Procedure: Ventilation/Perfusion SPECT with Galligas and 68Ga-MAA	• LEROUX Pierre-Yves, Brest, France
NCT00531180	Completed	No Results Available	• Esophageal Cancer • Lung Cancer	• Procedure: 4D CT scans • Procedure: Lung Function Imaging	• University of Texas MD Anderson Cancer Center, Houston, Texas, United States
NCT01200888	Terminated	No Results Available	• Cystic Fibrosis		• Stanford University School of Medicine, Stanford, California, United States
NCT05134558	Recruiting	No Results Available	• Lung Cancer • Radiation Therapy Complication	• Radiation: Xenon-enhanced Ventilation CT-guided Radiotherapy	• National Taiwan University Hospital, Taipei, Taiwan
NCT03357094	Unknown status	No Results Available	• Lung Neoplasms		• Tongren Hospital, Beijing, Beijing, China
NCT01034514	Terminated	No Results Available	• Lung Cancer	SPECT scanner and gamma camera, treatment planning system et al.	• Stanford University School of Medicine, Stanford, California, United States
NCT04702607	Recruiting	No Results Available	• Non-Small Cell Lung Cancer	• Procedure: contrast enhanced 4DCT	• Abamson Cancer Center of the University of Pennsylvania, Philadelphia, Pennsylvania, United States

### 3.4 Comparative study of CTVI and other images

Apart from CTVI, current research endeavors to explore diverse functional imaging modalities, particularly SPECT-based imaging that employs positron emission tomography (PET) to evaluate lung metabolism and ventilation. Additionally, MRI-based functional imaging is utilized, primarily leveraging magnetic resonance imaging (MRI) to visualize lung tissue motion and hemodynamics (10, 11).

#### 3.4.1 Comparative study with PET-based function imaging

PET-based functional imaging relies on the development of perfusion imaging techniques (28). It was shown that when combined with radioactive gas inhalation, PET-based imaging can roughly reflect lung blood flow and ventilation function, thereby better reflecting changes in local lung function during the course of radiotherapy (10). Using SPECT, HOOVER et al. (63) classified lung cancer patients into non-radioactive and radioactive pneumonia groups. They found that compared to the radioactive pneumonia groups, the mean lung dose is nearly 5Gy higher, and %V20Gy and %V30Gy are nearly 5% higher in the non-radioactive pneumonia groups. Meanwhile, MATUSZAK et al. (64) found that optimizing radiotherapy plans based on SPECT could reduce the mean lung dose, suggesting that SPECT-guided radiation plans can reduce the incidence and severity of radiation pneumonia.

**Figure 2:**
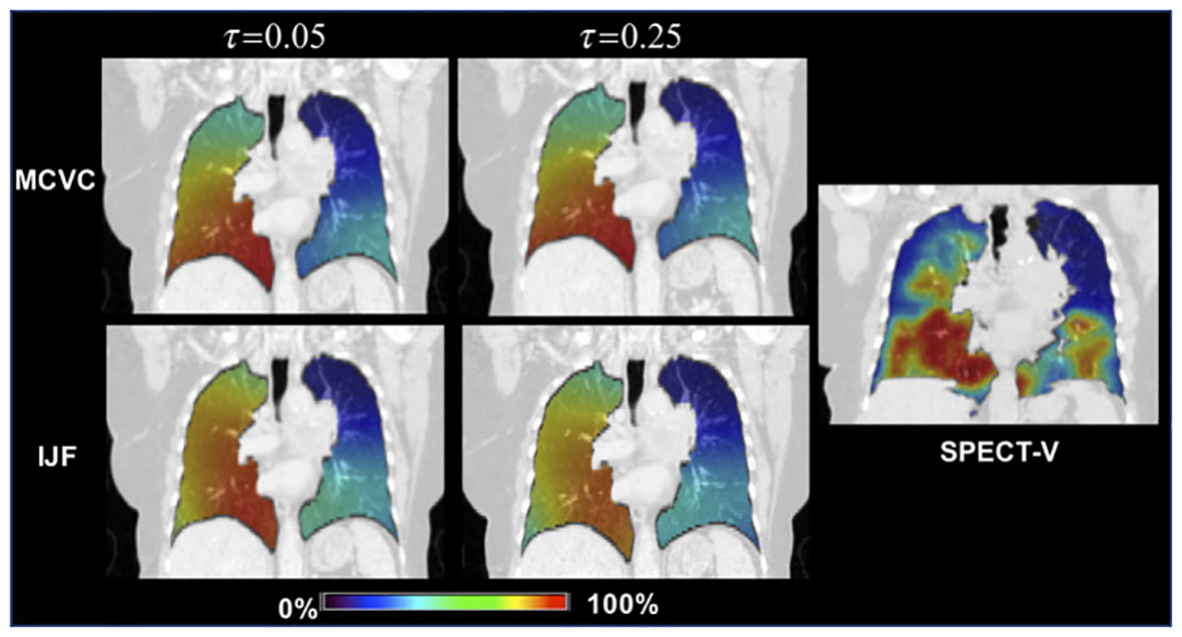
The correlation between 4D-CT and SPECT images Top Row: The 4DCT‐Inhale phase (left) and SPECT (right) ventilation images for the case with the lowest over correlation . Bottom Row: The IJF (left) and MCVC (right) superimposed on the 4DCT‐Exhale phase. Ventilation images were converted to percentile images for direct visual comparison. printed with permission from Castillo E, et al (65).

Castillo E et al. (65) investigated the correlation between CT ventilation-based lung functional imaging and SPECT perfusion in a study involving 15 pre-radiotherapy non-small cell lung cancer patients, comparing 4DCT and SPECT-V images. In each imaging case, CT-ventilation images utilizing Mass Conserving Volume Change (MCVC) and the Integrated Jacobian Formulation (IJF) were generated for 30 distinct uncertainty parameter values. They found that the median correlations between MCVC and SPECT-V ranged from 0.20 to 0.48 across the parameter sweep, while the median correlations for IJF and SPECT-V ranged between 0.79 and 0.82. The results indicated that robust methods generate ventilation images that are spatially consistent with SPECT-V, with the transformation-based IJF method yielding higher correlations than those previously reported in the literature (**Figure 2**).

#### 3.4.2 Comparative study with MRI

According to different techniques, MRI-based functional imaging mainly consists of MRI ventilation imaging, fluorinated gas MR imaging, pulmonary perfusion and hemodynamic imaging, as well as biomechanical evaluation for pulmonary functional imaging (66).

Hyperpolarized noble gas (3He or 129Xe) MR imaging, oxygen-enhanced MR imaging and fluorine-19 (19F) MR imaging have been studied as potential MR-based ventilation imaging techniques since the 1990s. They have been extensively tested for assessing disease severity and evaluating therapeutic effects of various pulmonary diseases (67–69). The pulmonary perfusion and hemodynamic imaging are widely used in visualizing pulmonary vasculature and blood flow (70, 71). The MR-based biomechanical assessment, currently, has been mainly attempted for radiation oncology rather than pulmonary functional imaging. Additionally, the absence of ionizing radiation renders these techniques suitable for experimental studies involving healthy subjects (72).

The correlation between MRI-based and CT-based functional imaging modalities in radiotherapy was investigated in a study conducted by Carey KJ et al. (73), which involved 34 patients undergoing both HP ³He MRI and CT imaging. Ventilation defects were evaluated using a semi-automated k-means clustering algorithm in HP ³He MRI. Parametric response mapping (PRM) was applied to inspiratory and expiratory CT images to quantify emphysema markers and indicators of functional small airways disease (fSAD). Results shown that fSAD was well correlated with the whole lung ventilation defect percent (VDP), whereas the correlation with forced vital capacity (FVC)%p was poor (-0.38 ≤ r ≤ -0.35, p < 0.001), as anticipated from previous studies. Furthermore, Matsumoto KI (74) proposed a novel methodology incorporating MRI, EPRI, and PET for investigating the tumour microenvironment. This research presents a novel concept that a multimodal instrument, such as PET-MRI, could potentially facilitate the integration of multiple functions.

The above functional imaging studies based on CTVI, SPETCT and MRI technology have their own characteristics showed in **Table 3**. SPECT perfusion imaging involves the use of 99mTc-labeled radioactive aerosols, which are hazardous and necessitate specialized aerosolization equipment. Furthermore, the image resolution of inert gas 133Xe is suboptimal. MRI using 3He faces cost constraints due to its limited source and the necessity of specialized inert gas polarization devices. Additionally, the inadequate signal and contrast derived from solid tissue and blood vessels hinder the accuracy of 3D MRI registration.

**Table 3:** The characters of CTVI, SPECT and MRI functional imaging.

Function imaging	CTVI (9, 16–18, 23–37, 39–58, 60–62)	SPECT (10, 17, 19, 38, 38, 59, 63–65, 75)	MRI (11, 15, 17, 38, 66–74)
Technique types	Ventilation	Ventilation/Perfusion	Ventilation/Perfusion/ Biomechanical assessment
Function Imaging qualities	1 Imaging resolution is depended by CT images, mostly high. 2 The imaging accuracy was effected by the DIR 3 Only reflect the ability of ventilation	1 The imaging resolution of inert gas 133Xe is not high. 2 The imaging accuracy was high 3 It could well reflect the ability of perfusion	1 Poor signal and contrast from solid tissue and blood vessels 2 The imaging accuracy was effected by the patient position (long scan time) and registration with CT (if applied to the radiotherapy) 3 It could well reflect the ability of perfusion
Patient safety	1 Patients is safety and easy to complete the scan 2 No added scan for radiotherapy patients 3 No additional radiation damage	1 Patients need to training before scan 2 Patients need additional scan and even inhalation radioactive gas 3 Additional radiation damage	1 Patients need to training before scan 2 Patients need additional scan and even inhalation gas 3 No additional radiation damage
Cost	No additional cost for radiotherapy patients	1 Needs special aeroso- lization equipment cost 2 SPECT scan needs high cost for patient 3 Radiopharmaceutical cost	1The 3He is expensive due to its source limitations 2 The requirement of specific inert gas polarization devices 3 MRI scan cost
Clinical value	1 It is easy to achieve and can widely be used in radiotherapy 2 Lots clinical trials about radiotherapy have been application 3 Prospective studies have demonstrated its clinical benefits in radiotherapy	1 It is not widely used in radiotherapy for the cost 2 Less clinical trials about radiotherapy have been application 3 No prospective studies have demonstrated its clinical benefits in radiotherapy	1 It is not widely used in radiotherapy, but widely used in pulmonary disease diagnosis 2 Less clinical trials about radiotherapy have been application 3 No prospective studies have demonstrated its clinical benefits in radiotherapy

### 3.5 Uncertainties in CT-ventilation

Ventilation function imaging utilizing CT technology occupies a pivotal position in radiomic oncology owing to its distinctive benefits, particularly its non-invasive nature and the absence of the need for extra CT scans. Research has further confirmed that dose-function metrics exhibit superior predictive power for radiation toxicity, in comparison to dose metrics alone, as evidenced by previous studies (58–62). Nonetheless, there remain several pertinent issues requiring attention, particularly uncertainties surrounding the principles, methods, and practical applications of ventilation function imaging.

#### 3.5.1 Uncertainties of principles

Pulmonary function evaluation involves assessing alveolar ventilation capacity, initially proposed by Geppert and ZIINtz in 1886 (76). Krogh further validated that pulmonary blood flow, oxygen absorption, and carbon dioxide elimination collectively affect gas exchange efficiency. He suggested describing local lung ventilation capacity by the ratio of ventilation-to-perfusion (77). This concept is now widely accepted in clinical and scientific research, emphasizing that lung function is influenced by both ventilation and perfusion. Consequently, CTVI, which only considers ventilation ability, cannot accurately depict gas exchange capability, i.e. the actual lung function. In recent years, there have been reports on establishing functional images based on ventilation perfusion by administering radioactive inhaled gas using dual-energy CT (40). However, research in this area remains limited. With the application of deep learning in functional imaging, it is expected that the uncertainty caused by CTVI only considering ventilation function can be reduced by using deep learning methods in the future.

#### 3.5.2 Uncertainties of methods

CTVI is primarily implemented through DIR. During 4DCT image acquisition, artifacts may arise due to irregular patient breathing and imaging limitations, impacting reliability and accuracy. Lung ventilation methods based on CT values are influenced not only by image registration results but also significantly by image quality (19). While Jacobian methods heavily rely on subjects’ breathing consistency in both breathing state and mode, the generated ventilation images are highly sensitive to the chosen DIR algorithm. Slight disturbances in DIR results can lead to substantial changes in ventilation estimation, resulting in poor model repeatability (75). The tissue-density method, independent of DIR, exhibits higher accuracy and reproducibility despite some limitations (38, 78). The method is invalid for tumor-blocked areas and areas exhibiting abnormal morphology, including lung tissues with fluid or surrounding tumors of HU values ≥-600. Importantly, deep learning methods have been proven to substantially improve the accuracy of ventilation images (43). Despite being in the research phase, these methods hold promise for overcoming the limitations of traditional methods.

#### 3.5.3 Uncertainties of applications

Numerous issues exist in the clinical application of pulmonary function. For instance, PET-based or MRI-based functional imaging requires additional scanning, and the cost of inhaled gas is prohibitive, limiting clinical application (11, 13, 33). While CT-based functional imaging does not encounter these issues, technical deficiencies (solely considering ventilation function, inaccurate pre-registration positions, etc.) necessitate further result validation (19, 65, 66). Additionally, changes in location of lung functional areas due to tumor regression during radiotherapy may result in lung function avoidance failure, aggravating dose exposure in some functional areas (62).

## 4 Summary and future research

The utilization of CT-based lung function imaging in radiation therapy holds considerable promise, albeit accompanied by persistent challenges. A crucial challenge lies in the lack of a standardized approach for evaluating the precision of lung function imaging. Furthermore, the imperative of ensuring the accuracy and reproducibility of functional parameters necessitates continued validation efforts. As such, future research endeavors must prioritize tackling these challenges and devising appropriate solutions.

A pivotal challenge for future research is to enhance the image quality and stability of CT imaging, as it is fundamental for precise assessment of lung tissue’s physiological and pathophysiological states. Researchers have extensively explored various methods, including techniques independent of image registration and deep learning approaches, to address this challenge. O. Ronneberger (48) highlights the effectiveness of FCN networks, particularly the U-Net, in leveraging limited annotated datasets through techniques like random elastic deformation for data augmentation. This approach enables the extraction of detailed image features without the need for additional training data, resulting in robust segmentation outcomes.

Secondly, the integration of CT perfusion imaging and CT elastography with additional imaging modalities, such as SPECT and MRI, can yield a more holistic evaluation of lung cancer patients’ condition and treatment efficacy. Combining CT-based lung ventilation imaging with PET and MRI-based blood perfusion imaging can enhance the depiction of gas exchange capacity in the functional lung, potentially establishing a standardized lung function image. Furthermore, recent research on dual-energy CT in conjunction with gas inhalation techniques (40) provides fresh perspectives on evaluating blood perfusion in the functional lung, hinting at groundbreaking paths for standardizing functional imaging protocols.

The assessment of CT-based lung function imaging technology’s efficacy in preventing and managing complications, particularly acute radiation pneumonia, and its role in guiding the development and modification of radiation therapy plans in clinical practice, is of paramount importance. Further investigation and research in these areas are warranted, as highlighted in previous studies (13, 28).

## 5 Conclusion

This study aim to shed light on the opportunities and challenges by a comprehensive examination encompassing the background, definition of relevant concepts, classification of existing studies, and review of pertinent literature in this burgeoning field. A comprehensive review of CTVI’s application in radiotherapy facilitates a thorough understanding of advancements and limitations of current research, providing valuable insights for advancing the field. Given the ongoing technological innovations, CT-based lung function imaging is anticipated to play a pivotal role in delivering individualized and precise radiotherapy to patients.
